# Individual variability in the anatomical distribution of nodes participating in rich club structural networks

**DOI:** 10.3389/fncir.2015.00016

**Published:** 2015-04-21

**Authors:** Madison Kocher, Ezequiel Gleichgerrcht, Travis Nesland, Chris Rorden, Julius Fridriksson, Maria V. Spampinato, Leonardo Bonilha

**Affiliations:** ^1^Department of Neurology and Neurosurgery, Medical University of South CarolinaCharleston, SC, USA; ^2^Department of Psychology, University of South CarolinaColumbia, SC, USA; ^3^Department of Communications Sciences and Disorders, University of South CarolinaColumbia, SC, USA; ^4^Department of Radiology, Medical University of South CarolinaCharleston, SC, USA

**Keywords:** connectome, magnetic resonance imaging, diffusion tensor imaging, structural networks, rich club, hub nodes

## Abstract

With recent advances in computational analyses of structural neuroimaging, it is possible to comprehensively map neural connectivity, i.e., the brain connectome. The architectural organization of the connectome is believed to play an important role in several biological processes. Central to the conformation of the connectome are connectivity hubs, which are likely to be organized in accordance with the rich club phenomenon, as evidenced by graph theory analyses of neural architecture. It is yet unclear whether rich club connectivity hubs are consistently organized in the same anatomical framework across healthy adults. We constructed the brain connectome from 43 healthy adults, based on T1-weighted and diffusion tensor MRI data. Probabilistic fiber tractography was used to evaluate connectivity between each possible pair of cortical anatomical regions of interest. Connectivity hubs were identified in accordance with the rich club phenomenon applied to binarized matrices, and the variability in frequency of hub participation was assessed node-wise across all subjects. The anatomical location of nodes participating in rich club networks was fairly consistent across subjects. The most common locations for rich club nodes were identified in integrative areas, such as the cingulate and pericingulate regions, medial aspect of the occipital areas and precuneus; or else, they were found in important and specialized brain regions (such as the oribitofrontal cortex, caudate, fusiform gyrus, and hippocampus). Marked anatomical consistency exists across healthy brains in terms of nodal participation and location of rich club networks. The consistency of connections between integrative areas and specialized brain regions highlights a fundamental connectivity pattern shared among healthy brains. We propose that approaching brain connectivity with this framework of anatomical consistencies may have clinical implications for early detection of individual variability.

## Introduction

Recent advances in neuroimaging now make it possible to chart the organization of neural connectivity across the entire human brain using magnetic resonance (MRI) diffusion tensor imaging (DTI) (Hagmann et al., 2008). Known as brain connectomes (Sporns et al., 2005; Sporns, 2011), the generation of whole brain maps of neural architecture is becoming increasingly popular as evidence accumulates in favor of the view that the disruption of structural connectivity is central to the neurobiology of many neurological and psychiatric illnesses (Buckner et al., 2009; Seeley et al., 2009; van den Heuvel and Sporns, 2011).

A common challenge encountered during connectome analysis is the identification of connectivity hubs, that is, nodes that are essential to the network, granting it a structured and non-random conformation. In general, hubs are expected to function as the basis of the brain's integrative capacity (van den Heuvel and Sporns, 2011; Sporns, 2013), and possess a high degree of connectivity, short neuronal path lengths, and high centrality (van den Heuvel and Sporns, 2011).

A promising strategy in identifying network hubs involves the assessment of nodal participation in so-called *rich club* sub-networks (Sporns, 2014). Assuming that central hubs of the connectome are more likely to be highly interconnected, rich-club nodes characteristically show higher connectivity with each other, beyond what would be expected by chance given their degrees (i.e., number of connections with other nodes).

Despite many reports demonstrating the presence of the rich club phenomenon in the brain (van den Heuvel and Sporns, 2011; van den Heuvel et al., 2012; van den Heuvel and Sporns, 2013; Collin et al., 2014) the actual anatomical variability of nodes pertaining to these kinds of networks across the healthy populations has yet to be established. Studies have so far demonstrated that the number and approximate locations of the hub nodes are similar (van den Heuvel and Sporns, 2011), while the exact position of each node differs slightly (Mueller et al., 2013). Thus, the normative definition of regional variability in rich-club nodes can aid the interpretation of future studies aiming to assess conformational differences in network architecture and hub sites, and their relationship with neurological and psychiatric diseases.

In this study, we aimed to measure the anatomical variability of nodal participation in structural rich club networks. We computed the whole brain connectome from a relatively large cohort of healthy individuals and we assessed the frequency with which anatomical sites were involved in the rich club. We hypothesized that, while the overall size of the rich club network (i.e., the number of nodes in the rich-club network) remained relatively stable across individuals, anatomical differences could be observed in its composition, for instance, in terms of nodal participation in rich club configuration.

## Methods

### Subjects

We studied 43 right-handed, healthy subjects [mean age ± standard deviation (SD) = 37.1 ± 11.7 years, 28 females] with no previous history of neurological or psychiatric disorders. The Institutional Review Board of the Medical University of South Carolina approved this study. All subjects signed an informed consent prior to their inclusion in the present study.

### MRI acquisition

All subjects underwent MRI scanning performed on a Verio 3 Tesla MRI scanner (Siemens Medical, Erlangen, Germany), yielding T1- and diffusion-weighted images (DWIs) obtained using a unique protocol across, as follows: (1) T1 weighted images: 3D magnetization-prepared rapid gradient echo (MPRAGE) sequences with parameters: repetition time (TR) = 2250 ms, echo time (TE) = 4.18 ms, flip angle = 6°, FOV = 256 × 256 mm, matrix size = 256 × 256, slice thickness: 1 mm and 192 sagittal slices; (2) DWI: twice-refocused, single-shot echo planar sequence with diffusion weightings *b*-value = 0, 1000, and 2000 s/mm^2^ applied along 30 non-collinear directions. Other imaging parameters were: *TR* = 8500 ms, *TE* = 98 ms, field of view = 222 × 222 mm, matrix size = 74 × 74, bandwidth = 1324 Hz/pixel, parallel imaging factor of 2, no partial Fourier encoding, number of excitations (NEX) = 10 for *b* = 0 s/mm^2^, and 1 for *b* = 1000, 2000 s/mm^2^, and 40 axial slices each 3 mm thick (no gap).

### Tractography and connectome measurement

White matter maps were obtained from segmentation of T1-weighted images using FreeSurfer (Martinos Center for Biomedical Imaging, Harvard-MIT, Boston USA) (Fischl et al., 2002). Gray matter regions of interest (ROI) were obtained by the segmentation of the cortex into 41 ROIs in each hemisphere in accordance with the Lausanne anatomical atlas, distributed as part of the Connectome Mapping Toolkit [http://www.cmtk.org/] (listed in Table 1). Gray matter ROIs and white matter maps were transformed onto the image space of DWIs through linear registration using FSL FLIRT (Xue et al., 1999; Jenkinson et al., 2002).

**Table 1:** **Percent nodal participation in the rich club for healthy participants recruited in this study based on networks obtained with a fixed density threshold in the 95th percentile**.

**Number**	**Node**	**Rich club participation (%)**
1	***Right lateral occipital***	***90.69767442***
2	***Right pericalcarine***	***88.37209302***
3	***Right caudal anterior cingulate***	***86.04651163***
4	***Left posterior cingulate***	***86.04651163***
5	***Right posterior cingulate***	***83.72093023***
6	***Right lateral orbitofrontal***	***83.72093023***
7	***Right fusiform***	***83.72093023***
8	***Right caudate***	***83.72093023***
9	***Left supramarginal***	***83.72093023***
10	***Left lateral orbitofrontal***	***83.72093023***
11	***Left inferior temporal***	***83.72093023***
12	***Right middle temporal***	***81.39534884***
13	***Right lingual***	***81.39534884***
14	***Right hippocampus***	***81.39534884***
15	***Left precuneus***	***81.39534884***
16	***Left hippocampus***	***81.39534884***
17	***Left caudate***	***81.39534884***
18	***Left caudal middle frontal***	***81.39534884***
19	Right isthmus cingulate	79.06976744
20	Left middle temporal	79.06976744
21	Right pars opercularis	76.74418605
22	Right inferior parietal	76.74418605
23	Left rostral middle frontal	76.74418605
24	Left post central	76.74418605
25	Left lingual	76.74418605
26	Left inferior parietal	76.74418605
27	Left superior temporal sulcus	76.74418605
28	Left amygdala	76.74418605
29	Right supramarginal	74.41860465
30	Right superior temporal	74.41860465
31	Right precuneus	74.41860465
32	Right postcentral	74.41860465
33	Right caudal middle frontal	74.41860465
34	Right superior temporal sulcus	74.41860465
35	Left superior temporal	74.41860465
36	Left pars opercularis	74.41860465
37	Left insula	74.41860465
38	Left fusiform	74.41860465
39	Right rostral middle frontal	72.09302326
40	Right rostral anterior cingulate	72.09302326
41	Right medial orbitofrontal	72.09302326
42	Right accumbens	72.09302326
43	Left medial orbitofrontal	72.09302326
44	Left caudal anterior cingulate	72.09302326
45	Right inferior temporal	69.76744186
46	Left rostral anterior cingulate	69.76744186
47	Left pericalcarine	69.76744186
48	Left pars triangularis	69.76744186
49	Left lateral occipital	69.76744186
50	Left isthmus cingulate	69.76744186
51	Right superior parietal	67.44186047
52	Right pars triangularis	67.44186047
53	Right pallidum	67.44186047
54	Right amygdala	67.44186047
55	Left pallidum	67.44186047
56	Right superior frontal	65.11627907
57	Left thalamus	62.79069767
58	Right thalamus	60.46511628
59	Right temporal pole	60.46511628
60	Right insula	60.46511628
61	Left superior frontal	60.46511628
62	Right parahippocampal	58.13953488
63	Right paracentral	58.13953488
64	Left superior parietal	55.81395349
65	Right pars orbitalis	51.1627907
66	Right cuneus	51.1627907
67	Left temporal pole	51.1627907
68	Left parahippocampal	51.1627907
69	Left cuneus	51.1627907
70	Right precentral	48.8372093
71	Left pars orbitalis	48.8372093
72	Left accumbens	48.8372093
73	Left paracentral	46.51162791
74	Left precentral	39.53488372
75	Right entorhinal	20.93023256
76	Left frontal pole	20.93023256
77	Right frontal pole	18.60465116
78	Left entorhinal	18.60465116
79	Left transverse temporal	16.27906977
80	Right putamen	6.976744186
81	Right transverse temporal	4.651162791
82	Left putamen	2.325581395

*Nodes represent each of the 82 regions of interest derived from the gray cortical map. Rich club participation was defined as those nodes with degree j such that Φ(j)/Φ^random^(j) > 1. Structures in bold were rich club nodes in at least 80% of the participants*.

Whole brain tractography was reconstructed in DWI space. Tractography was performed using the software Diffusion Toolkit (DTK) (Wang et al., 2007). The acquisition geometry and gradients were obtained from DICOM images using dcm2nii, as part of the software MRIcron (Rorden et al., 2007). The parameters for tractography were as follows: (1) maximal angle threshold of 45° (Mori and van Zijl, 2002); (2) inclusion mask from dynamic contrast range mask based on the diffusion-weighted image (DWI), as part of default DTK parameters; (3) inclusion mask from white matter map (in DWI space). Every white matter fiber was evaluated regarding its extreme points. If the extreme point of the fiber was located in the boundary between gray and white matter region (i.e., within approximately one or fewer voxels from the gray matter ROI), this extreme was considered to be linking this ROI. If both extremes were linking different gray matter ROIs, the fiber was counted as connecting these ROIs. For each possible pair of ROIs, the number of fibers connecting the pair was computed and recorded in a connectivity matrix. The connectivity was corrected due to the biases arising from the length of streamlines and volume sizes of the different ROIs (Hagmann et al., 2007). These steps were performed through in-house scripts written in MATLAB.

For each subject, the resulting connectome was represented as a weighted connectivity matrix ***A***, symmetrical along its main diagonal (i.e., undirected), where each entry ***Aij*** corresponded to the weighted link between ROIs ***i*** and ***j***.

### Calculation of the rich club network

For each subject, the nodes participating in the rich club network were defined as those with high degree, which were more densely connected with other highly-interconnected nodes than it would be expected just by chance based on their high degree (Sporns, 2013, 2014). We employed the same methodological approach previously described by Sporns (Sporns, 2013) in order to identify such rich-club nodes. We binarized each individual network based on a range of fixed density thresholds, whereby all links with weight higher than 60th to the 95th percentile of individual weight distribution were maintained. This step maintained only the reproducible core of the network, which was associated with high number of streamlines in each subject.

For each fixed density threshold, the rich club coefficient was iteratively calculated across all degrees (k) observed in the network, as the sum of connections of the sub-network composed of nodes with higher degrees (>k), divided by the number of all possible connections within this sub-network. For each network degree, the rich club coefficient is denoted as Φ, and a corresponding vector is obtained for each subject representing the coefficient across all degrees, Φ (k). A normalized coefficient was then obtained by calculating the element by element ratio between Φ(k) and Φ^random^(k), where Φ^random^(k) denotes the rich club coefficient calculated (also across the range k) for 1000 random networks obtained by shuffling the links in the individual network, preserving the degree distribution compared with the individual network. For each subject, nodes participating in the rich club network were defined as those with degree *k* such that Φ(k)/Φ^random^(k) > 1.

We examined the variability across subjects of nodal participation in the rich club network by evaluating the relative frequency with which each node was part of the rich club network. We compared regional participation in the rich club networks across all fixed density thresholds through a series of chi-squares. Nonetheless, we focused our analyses on the data obtained from the networks including only links within the highest 5th percentile of link weight, which is a sparsity level more likely to represent the core framework of structural organization of the networks obtained with probabilistic tractography (Hinne et al., 2015).

Finally, we also evaluated whether there was a correlation between the frequency of participation in the rich club network and age.

## Results

There were no statistically significant differences in regional rich club participation when comparing different fixed density thresholds. The relative participation of nodes in the rich club network across all thresholds can be observed in Supplementary Figure 1.

**Figure 1:**
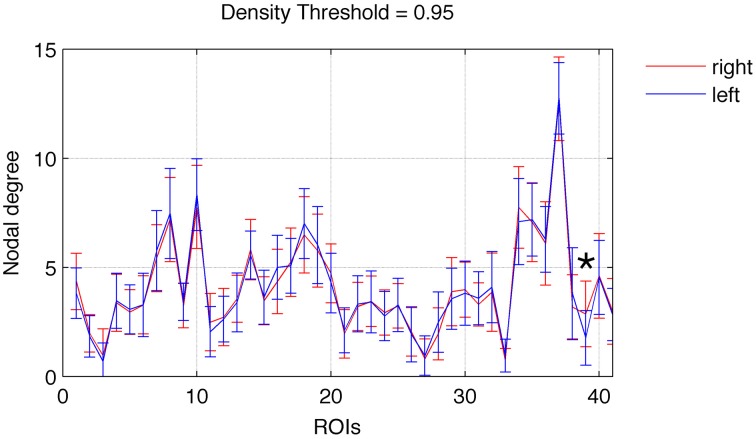
**Nodal degree (mean ± SD) for each ROI in the left (blue) and right (red) hemispheres**. A significant difference between the right and left hemispheres was found exclusively for ROI 39 (nucleus accumbens), marked with an asterisk. Error bars are SD.

Focusing on networks composed of links with weight greater than the 95th percentile (in order to better represent network cores) we observed a relatively left vs. right symmetrical distribution of nodal degrees. As demonstrated in Figure 1, there was not a statistically significant difference in node-wise distribution of degrees across hemispheres, except for the nucleus accumbens (ROI 39), which was associated with a higher degree on the right hemisphere (*t* = 3.98, *p* = 0.0003).

The hemispheric distribution of node-wise rich club participation was also fairly symmetrical across hemispheres. Nonetheless, the lateral occipital region was more commonly part of the rich club network in the right (*t* = 2.95, *p* = 0.005). Notably, employing a more liberal statistical threshold, (i.e., 0.01 < p < 0.05), we observed a trend toward higher participation of the right pericalcarine region (*t* = 2.07, *p* = 0.04), the right nucleus accumbens (*t* = 2.67, *p* = 0.01), and the left transverse temporal region (*t* = −2.35, *p* = 0.02).

**Figure 2:**
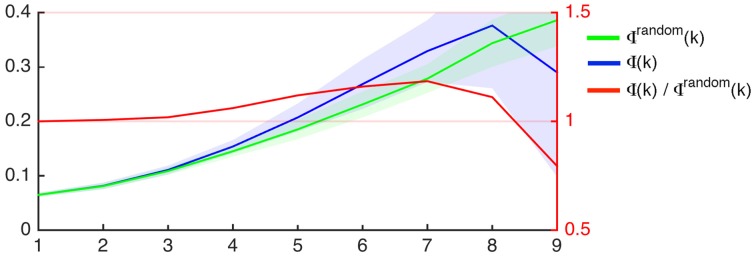
**Rich club coefficient as a function of nodal degree (x-axis) for the average connectome of participants in this study (Φ, blue) and for 1000 random networks with similar degree distribution (Φ^**random**^, green)**. The left Y-axis demonstrates the rich club coefficient. The shaded areas represent the interval within 1 SD above and below the mean, which is demonstrated by the continuous colored line. The red line represents the proportion between Φ and Φ^random^ as a function of degree (X-axis), and the ratio is demonstrated on the right Y-axis.

Group wise average curves demonstrating Φ (k) (i.e., the rich club coefficient per degree k), Φ^random^(k), and their ratios are shown in Figure 2. As described in the methods, the criterion for defining nodes participating in the rich club network is defined as Φ (k)/Φ^random^(k) > 1. The nodal degrees where the ratio Φ (k)/Φ^random^(k) was greater than 1 was variable among subjects, as demonstrated in Supplementary Figure 2, which demonstrates Φ (k), Φ^random^(k), and Φ(k)/Φ^random^(k) for all subjects.

The average number of nodes pertaining to the rich club, per subject, was 45 ± 13 nodes. A chi-square comparison of the observed versus expected frequencies for each node demonstrated that the following nodes were more likely to be involved in the rich club network: right lateral occipital, right pericalcarine, right caudal anterior cingulate, left posterior cingulate, right posterior cingulate, right lateral orbitofrontal, right fusiform, right caudate, left supramarginal, left lateral orbitofrontal, left inferior temporal, right middle temporal, right lingual, right hippocampus, left precuneus, left hippocampus, left caudate, left caudal middle frontal regions.

**Figure 3:**
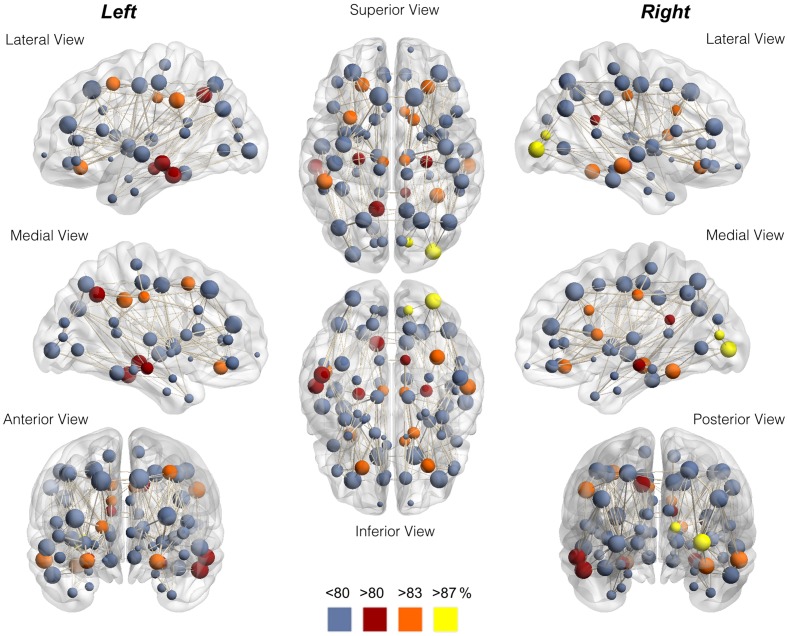
**Anatomical distribution of nodes color-coded for node-wise percentage of participation in the rich club**. Notice the preferential location in the proximity of medial integrative areas, medial aspect of occipital and pre-cuneus regions, and highly specialized brain regions. The data in this figure corresponds to Table 1.

The node-wise percentage of participation in the rich club for each node is demonstrated anatomically in Figure 3 and in Supplementary Figure 3. The individual variability can also be appreciated in Supplementary Figure 4, which demonstrates the nodes participating in the rich club network for each individual assessed in this study.

We did not observe a relationship between age and the number of nodes pertaining to the rich club network. However, we observed a trend toward a higher participation of the left entorhinal cortex in the rich club network among older individuals (i.e. older than the median age in our sample, 35 years), Chi^2^ = 9.382, *p* < 0.005.

## Discussion

In this study, we demonstrated that nodes participating in rich club networks are consistently located in relatively equivalent anatomical locations across healthy adults. We also observed that the rich club nodes are located on all brain lobes in a fairly symmetrical distribution either in their medial or lateral aspects.

Interestingly, the anatomical areas more commonly associated with rich club nodes were those located in the proximity of medial integrative areas, such as the cingulate and pericingulate regions, medial aspect of the occipital areas and precuneus; or else, they were found in specialized brain regions (such as the oribitofrontal cortex, caudate, fusiform gyrus and hippocampus). These results suggest that these areas not only play an important functional role, but they also play an important part in the configuration of the structural brain networks in healthy individuals (Sporns, 2013). Specifically, they may serve as structural hubs and provide the structural framework for integration across different functional domains (Sporns, 2014). Our observations are in accordance with the theory that informational processing associated with a non-randomly and consistently organized structural pattern (van den Heuvel et al., 2012).

We observed mild hemispheric asymmetry in regional rich club participation due to a higher prevalence of the right nucleus accumbens. This finding may be related to functional specialization of the nucleus accumbens in motor (Budilin et al., 2008), behavioral control (Savjani et al., 2014) and/or learning and memory (Downar et al., 2011). Furthermore, the higher participation of the entorhinal cortex in the rich club networks related to aging may be signifying increased medial temporal lobe network centrality in relationship with compensatory aging memory processing (Du et al., 2003).

In this study, we employed an atlas featuring 82 ROIs, since this parcellation method is commonly used in the neuroimaging literature (Hagmann et al., 2008) and our results can be related to other studies employing a similar approach. Nonetheless, it is yet unknown whether cortical parcellation methods exert a significant influence on the ensuing network architecture. In theory, a higher number of nodes could lead to a better evaluation of finer grained network complexity. With current limitations in spatial resolution of DTI, building the connectome with smaller ROIs can lead to an inability to resolve micro-connectivity.

Furthermore, our results are related to structural networks constructed using probabilistic tractography and should be interpreted in this context. Probabilistic tractography is a commonly used approach to evaluate DTI data, with the ability to partially resolve complex fiber anatomy and fiber crossings (Behrens et al., 2007). The streamline count obtained from probabilistic tractography is considered to be a biophysical representation of the underlying axonal bundles and myelin sheath (Jones et al., 2013). However, since the calculation of tractography streamlines is assessed from the distributions on voxel-wise principal diffusion directions, there is an inherent possibility that noise or low probability connections are included in the connectome. For this reason, the evaluation of networks based on density thresholds permits a more accurate assessment of the core framework of connectivity. Thus, we used the number of streamlines as a binary threshold method—i.e., regional links with a very high number of fibers were likely to be considered a core in the connectome framework, and therefore maintained in the network analyses. This “fixed density threshold” approach does not use number of fibers as weighted links in the network in the subsequent rich club analyses. We focused on a 95th percentile threshold to maintain consistency with the methodology from previous studies (van den Heuvel and Sporns, 2011; Cammoun et al., 2012; Hinne et al., 2015). Nonetheless, our exploratory analyses using different fixed density thresholds demonstrate a fair reproducibility in regional rich club participation across multiple sparsity levels.

There are many potential and practical implications from observations from this study, especially with regard to gain or loss of hub function by brain regions in specific pathological or developmental processes. For example, while studies to date have evaluated the number of rich club networks in particular disease states (e.g., see van den Heuvel and Sporns, 2011), it is conceivable that positioning of hubs may also be affected by network rearrangement as a consequence of neuropathological or neurodevelopmental processes. This may also aid in the further characterization of phenotypical manifestations of neurological diseases, as, for example, different network rearrangements may be associated with neurological or psychiatric symptoms. Thus, by better evaluating the plastic changes associated with neural architecture organization, it may be possible to correctly identify biomarkers related to neurological and neuropsychiatric problems. As an example, different neural networks may be involved in the generation and maintenance of seizures in patients with temporal lobe epilepsy (Bonilha et al., 2012). However, the clinical and electrophysiological manifestations of epilepsy may be indistinguishable regarding the neuronal networks that originate seizures. This is clinically relevant, as response to treatment of epilepsy may be directly associated with the underlying neurobiological mechanisms of seizure generation and propagation (Bonilha et al., 2013). By evaluating changes in core configuration of temporal lobe network hubs, it may be possible to better identify the conformation of different phenotypes of temporal lobe epilepsy, and to potentially contribute with similar approaches to other pathological models.

Another example of direct application of anatomical positioning of rich club nodes is the evaluation of loss and reestablishment of hubs in relationship with brain injury. For example, it is commonly observed that subjects with language processing problems (aphasia) after cortical and subcortical injury after strokes may recover spontaneously or with speech therapy (Holland et al., 1996; Robey, 1998). It is commonly assumed that recovery involves the re-establishment of new networks to support the regaining of function. Nonetheless, it is still undefined whether a greater loss of network hubs may actually prevent a more full recovery. Similarly, it is unclear whether recovery may be associated with the reclaiming of hub status by areas that are typically not associated with the rich club.

In this study, we aimed to provide a description of the anatomical positioning and variability of nodes participating in rich club networks. The findings from this study may provide a reference and framework for future studies that assess changes in this pattern in association with neurobiological mechanisms related to health and disease.

### Conflict of interest statement

The authors declare that the research was conducted in the absence of any commercial or financial relationships that could be construed as a potential conflict of interest.

## Supplementary material

Supplementary Figure 1 **Percent nodal participation in the rich club for healthy participants recruited in this study based on networks obtained with fixed density thresholds ranging from 60th to the 95th percentile**. When correcting for multiple comparisons there we no differences in participations across thresholds. Using a less conservative approach (i.e., not correcting for multiple comparisons, with *p* < 0.05), we observed lesser rich club participation of the R posteriorcingulate, R postcentral, R/L fusiform, L caudalanteriorcingulate, L bankssts (superior temporal sulcus) below the threshold set 90th percentile; lesser rich club participation of the R pericalcarine below 95th percentile, lesser rich club participation of the L paracentral, L caudalanteriorcingulate and L cuneus from 90th to 95th percentiles. (R, right hemisphere; L, left hemisphere).

Supplementary Figure 2 **This figure provides a demonstration of individual rich club coefficients as a function of nodal degree (x-axis) (Φ, blue) and for 1000 random networks with similar degree distribution (Φ^random^, green)**. The left y-axis demonstrates the rich club coefficient. The red line represents the proportion between Φ and Φ^random^ as a function of degree (x-axis), and the ratio is demonstrated on the right y-axis.

Supplementary Figure 3 **This figure provides an anatomical reference for the nodes color-coded for node-wise percentage of participation in the rich club**. The data in this figure is similar to Figure 3, but all nodes are numbered in accordance with Table 1 to facilitate visualization.

Supplementary Figure 4 **This figure demonstrates the location of nodes participating in the rich club network (yellow) for all subjects assessed in this study**. Nodes not participating in the rich club network are demonstrated in dark red.
