# Supplementary figures and images for: Individual variability in the anatomical distribution of nodes participating in rich club structural networks
Source: Front Neural Circuits. 2015 Apr 21;9:16. doi: 10.3389/fncir.2015.00016 (PMC4405623; doi:10.3389/fncir.2015.00016)

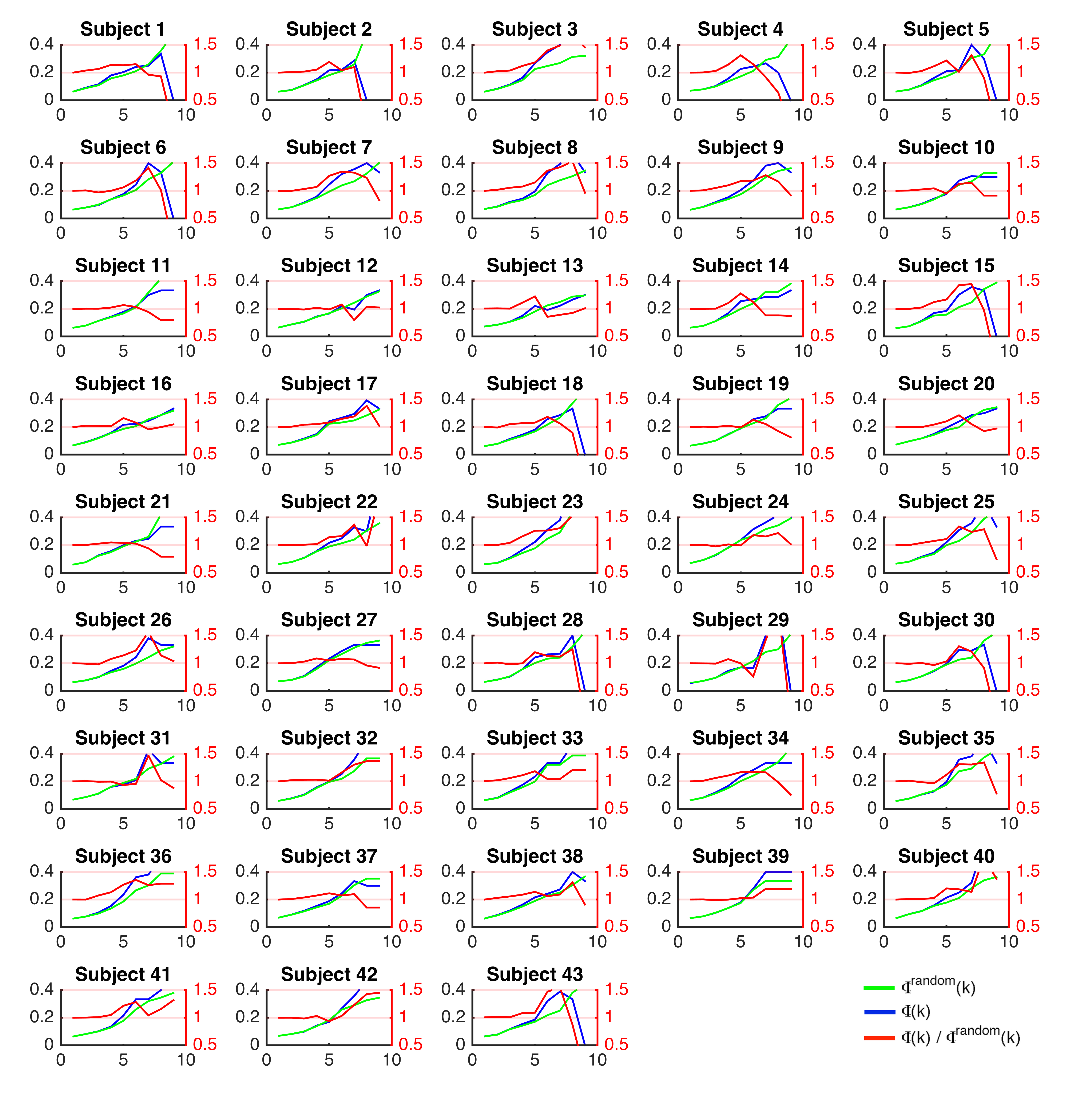

Supplement: Supplementary Figure 2 — This figure provides a demonstration of individual rich club coefficients as a function of nodal degree (x-axis) (Φ, blue) and for 1000 random networks with similar degree distribution (Φrandom, green). The left y-axis demonstrates the rich club coefficient. The red line represents the proportion between Φ and Φrandom as a function of degree (x-axis), and the ratio is demonstrated on the right y-axis. [file Image2.JPEG]
